# Breathing Biofeedback as an Adjunct to Exposure in Cognitive Behavioral Therapy Hastens the Reduction of PTSD Symptoms: A Pilot Study

**DOI:** 10.1007/s10484-015-9268-y

**Published:** 2015-03-07

**Authors:** A. Rosaura Polak, Anke B. Witteveen, Damiaan Denys, Miranda Olff

**Affiliations:** 1 Department of Anxiety Disorders, Academic Medical Center (AMC), University of Amsterdam (UvA), Meibergdreef 5, 1105 AZ Amsterdam, The Netherlands; 2 The Netherlands Institute for Neuroscience (NIN), Royal Netherlands Academy of Arts and Sciences, Meibergdreef 47, 1105 BA Amsterdam, The Netherlands; 3 Arq Psychotrauma Expert Group, Nienoord 5, 1112 XE Diemen, The Netherlands

**Keywords:** Posttraumatic stress disorder (PTSD), Breathing biofeedback, Trauma-focused cognitive behavioral therapy (TF-CBT), Working memory

## Abstract

Although trauma-focused cognitive behavioral therapy (TF-CBT) with exposure is an effective treatment for posttraumatic stress disorder (PTSD), not all patients recover. Addition of breathing biofeedback to exposure in TF-CBT is suggested as a promising complementary technique to improve recovery of PTSD symptoms. Patients (n = 8) with chronic PTSD were randomized to regular TF-CBT or TF-CBT with complementary breathing biofeedback to exposure. PTSD symptoms were measured before, during and after TF-CBT with the Impact of Event Scale-Revised. The results show that breathing biofeedback is feasible and can easily be complemented to TF-CBT. Although PTSD symptoms significantly decreased from pre to post treatment in both conditions, there was a clear trend towards a significantly faster (*p* = .051) symptom reduction in biofeedback compared to regular TF-CBT. The most important limitation was the small sample size. The hastened clinical improvement in the biofeedback condition supports the idea that breathing biofeedback may be an effective complementary component to exposure in PTSD patients. The mechanism of action of breathing biofeedback may relate to competing working memory resources decreasing vividness and emotionality, similar to eye movement desensitization and reprocessing. Future research is needed to examine this.

## Introduction

With 80 % of the general Dutch population experiencing a traumatic event once in their life (de Vries and Olff 2009) and 7–9 % developing a posttraumatic stress disorder (PTSD; Kessler et al. 1995; de Vries and Olff 2009) optimal treatment should be available for patients suffering from this disorder. Until now, “trauma-focused” treatments, such as eye movement desensitization and reprocessing (EMDR) and trauma-focused cognitive behavioral therapy (TF-CBT) have been shown to be equally effective in reducing PTSD symptoms (Bisson and Andrew 2007; Nijdam et al. 2012; Bradley et al. 2005; Seidler and Wagner 2006). One of the key elements in TF-CBT, is prolonged exposure (PE), in which patients are asked to relive their trauma by telling about it in detail.

Although TF-CBT and EMDR both can reduce traumatic stress symptoms, not all patients recover (Bisson and Andrew 2007). Furthermore, previous studies show significant rates of non-response (Schottenbauer et al. 2008) and drop-out (Nijdam et al. 2012; Schnurr et al. 2007). This may relate to difficulties in fully engaging during exposure, due to resistance of the patient to become highly distressed, or even engaging in therapy (not showing up during the sessions or eventually drop-out). As engaging during the session is essential for exposure to be efficacious and for habituation to occur, it may be helpful to add complementary elements or techniques, that may decrease resistance and increase tolerance to distress.

Biofeedback treatment may be a promising complementary treatment of CBT to further improve PTSD symptom recovery. During biofeedback, patients receive feedback from changes in physiological activity (i.e., heart rate variability or breathing). This technique can be implemented in treatment as usual. For example, breathing biofeedback as an adjunct to imaginal exposure therapy may entail breathing at a certain pace during imagining the traumatic event. By adding a biofeedback component, such as breathing biofeedback, exposure treatment may be better manageable for PTSD patients and result in better engagement. Especially during the first sessions, when symptoms may temporarily increase, adding this breathing component during exposure may decrease distress and induce engagement, consequently resulting in habituation. Another possibility is that additional breathing biofeedback is a competing working memory resource that consequently leads to less vivid and emotional images and thereby decreases PTSD symptoms, similarly as shown in other tasks taxing working memory (van den Hout et al. 2011a, b).

The effects of various types of biofeedback as an adjunct to regular psychological treatment have previously been investigated. For example, studies of depressed patients reported a reduction in depressive symptoms (Karavidas et al. 2007) as well as anxiety symptoms (Siepmann et al. 2008; Karavidas et al. 2007). Particularly in anxiety disorders, for which physiological arousal is eminent, an increasing number of studies report positive results of various forms of biofeedback added to CBT, e.g. heart rate variability (HRV) biofeedback and respiratory sinus arrhythmia (RSA) in panic disorder (Meuret et al. 2004) and particularly in PTSD (Gevirtz and Dalenberg 2008; Morina et al. 2012).

Previous RCTs in PTSD patients have shown to be effective in decreasing PTSD specific symptoms (Tan et al. 2011) as well as comorbid depressive symptoms (Zucker et al. 2009) with additional HRV and RSA biofeedback treatment, respectively. Additional beneficial effects of biofeedback over and above the effects of CBT alone, were however not confirmed in another trial (Lande et al. 2010) since there was no significant difference in the decrease of PTSD symptoms between the HRV biofeedback condition and treatment as usual. Thus far, results are limited and inconclusive. In these previous studies, the biofeedback procedure was offered by separate and more time consuming extra sessions with biofeedback, though it would be more informative to examine feasibility and the course of PTSD symptoms when biofeedback is implemented directly into the session with the treatment as usual. This type of research would add useful information and may be helpful in drawing further conclusions on the effectiveness of biofeedback adjunct to CBT.

The aim of this pilot study, therefore, was to investigate the feasibility of breathing biofeedback when it is implemented as a direct adjunct to the exposure element within CBT sessions and to examine whether this leads to a significant pre to post treatment decrease of PTSD symptoms. By comparing the regular TF-CBT with the TF-CBT + biofeedback treatment, we aim to explore whether the addition of breathing biofeedback to exposure in CBT, further modifies symptom reduction by promoting engagement or distraction during exposure.

Based on previous studies (Zucker et al. 2009; Lande et al. 2010), we hypothesize that both breathing biofeedback and treatment as usual will lead to a significant decrease of PTSD symptoms when compared to pre treatment PTSD symptoms. Most interestingly, we expect a difference between conditions on PTSD symptom reduction, i.e., larger PTSD symptom reduction in the biofeedback condition than regular TF-CBT condition, as is consistent with a previous study (Tan et al. 2011).

## Methods

### Participants

Patients with chronic PTSD from an outpatient clinic of the Academic Medical Center (AMC) were invited to participate in the study. PTSD was diagnosed by an experienced clinician using the Clinician-Administered PTSD Scale (CAPS; Blake et al. 1995). Patients with a CAPS score of 45 or more were included in the study. Patients were excluded when other axis I disorders were present using the Mini-International Neuropsychiatric Interview (M.I.N.I-Plus; Sheehan et al. 1998). The Hamilton Depression Rating Scale (HDRS; Hamilton 1960) and clinical impression of the medical expert were used to measure the severity of comorbid depression. Patients were excluded in case of comorbid severe depressive disorder and when additional pharmacotherapy to TF-CBT was indicated. Patients taking psychotropic drugs were also excluded. After informed consent, patients were randomized to either the control condition in which they received treatment with TF-CBT or to the biofeedback condition in which exposure similar to the regular TF-CBT group was combined with breathing biofeedback. TF-CBT consisted of weekly sessions with exposure as the key element. The biofeedback group also received TF-CBT with breathing biofeedback complemented to exposure. Both groups received therapy sessions given by therapists that were part of the clinical workers of our department. They all had clinical experience and at least a Master’s degree in Clinical Psychology. Furthermore, they all had followed a training for TF-CBT at our department, in order to properly execute the clinician manual. The therapists received supervision regularly (once a month).

Of the nine patients that met the in- and exclusion criteria, eight (6 females and 2 males) were included (one patient declined). Participants experienced single-event traumata such as motor vehicle accidents, sexual or physical abuse. Their median age was 45 years (with a range of 25–57). Total number of sessions over time differed within the sample, median number of sessions was 7.5 (with a range between 5 and 18 sessions), of which all but one patient responded within the range of 8–12 sessions, consistent with the guidelines of CBT (Creamer et al. 2004).

### Materials and Procedure

All patients were randomized to treatment as usual consisting of TF-CBT (TAU condition) or to TF-CBT with an adjunct of biofeedback (biofeedback condition). TF-CBT was based on the model original developed by Foa et al. (1995) for female victims of rape. For TF-CBT, a strict protocol was used (Creamer et al. 2004), that included exposure (imaginal and in vivo) as its key element. Other elements were psycho-education and anxiety management. For more details concerning the protocol, see elsewhere (Polak et al. 2012). When randomized to the biofeedback condition, the breathing biofeedback device was introduced during the first (psycho-education) session. The introduction consisted of instructions for the use and practice during the session. A homework assignment following the first session included every day practice with the breathing biofeedback device when in relaxed state, which was expected to be sufficient to get comfortable with the device in order to be able to use the device during imaginal exposure in the sessions. From session three on (concurrent with start of focusing on hotspots, i.e., one or more emotional climaxes in the event that evoked the most fear or other emotional arousal) patients were instructed to use breathing biofeedback when focusing on a hotspot during imaginal exposure.

The biofeedback device selected for the study was the RespiFit and was provided by the University of Amsterdam (UvA) and the Dutch Institute for Scientific Research and Breathing Regulation and Health Advancement (Nederlands Instituut voor Wetenschappelijk Onderzoek naar Ademregulatie en Gezondheidsbevordering; NIAG). The breathing device measured the breathing frequency per minute. As mentioned previously, the breathing biofeedback device was introduced during the first (psycho-education) session. The introduction consisted of instructions for the use and practice during the session in order to familiarize the patient with the breathing component. The breathing frequency was programmed in advance. The device provided immediate feedback by a beep sound through an earplug. The device monitored whether the breathing followed the rhythm of the programmed breathing frequency. When the breathing frequency did not follow the breathing frequency anymore, sounds were offered as a sign that the patient should adjust the breathing frequency. When patients were breathing following the exact breathing rhythm, no sounds were offered anymore. During the actual therapy sessions, the procedure of the biofeedback device was implemented in the exposure element of TF-CBT and used when focusing on a traumatic hotspot. This procedure was similar for all patients.

Feasibility was assessed based on the ease of instructing patients and implementing it in the session. Furthermore, treatment adherence was assessed by keeping a record of attendance to the sessions, completion of homework assignments as well as treatment completion.

The main outcome measure consisted of the Impact of Event Scale-Revised (IES-R; Weiss and Marmar 1997) to measure PTSD symptoms. The IES-R is a 22-item self-report measure (with five point Likert scales, 0–4) that assesses subjective distress caused by traumatic events and is known as a solid measure of posttraumatic symptoms that is used often in clinical as well as research settings (Beck et al. 2008). Items correspond directly to 14 of the 17 DSM-IV symptoms of PTSD and all three symptom clusters [re-experiencing (8 items, scale range 0–32), avoidance (8 items, range 0–32) and hyper arousal (6 items, range 0–24)]. High levels of internal consistency have been previously reported (intrusion: Cronbach’s alpha = .87–.94, avoidance: Cronbach’s alpha = .84–.87, hyper arousal: Cronbach’s alpha = .79–.91; Weiss and Marmar 1997). Test–retest reliability, collected across a 6-month interval, ranged from .89 to .94 (Weiss and Marmar 1997). The IES-R was assessed at baseline (i.e., pre treatment), at each weekly session and at follow-up (i.e., 1 week post treatment).

The number of sessions was based on the Subjective Units of Distress (SUDS), a rating system on a 100-point scale ranging from 0 (no anxiety) to 100 (extreme anxiety). When the SUDS appeared to have dropped (below 0–30), showing that the patient did not experience extreme anxiety anymore while imagining the traumatic event, the therapy was completed.

### Statistical Analyses

Baseline differences on demographic and clinical characteristics between the biofeedback group and the TAU group were calculated using the non-parametric Mann–Whitney U-test for continuous variables. Fisher’s exact tests for small samples were used to assess ordinal variables.

Linear mixed-effects models with restricted maximum likelihood estimation and a fixed effect intercept were used to test whether the conditions (biofeedback and TAU) showed a differential effect on PTSD symptoms (IES-R) from pre to post treatment assessment. We also tested a mixed model with a Time factor consisting of the weekly IES-R scores instead of only the pre and post treatment assessment (T0 and T1). Although some patients received more sessions, we used 9 sessions in the model, as the majority of patients did not need more than 9 sessions. A mixed model is preferable over repeated measures ANOVA because it can handle missing values and measurements taken at unequival intervals. Thus, missing data were not replaced but handled in the mixed model. The mixed models included Time, Condition and an interaction between Time and Condition as within-subjects factors. The error structure of repeated measures was modeled as AR1 because correlation between measurements decreases as time points get further apart. We controlled for the baseline measurement (T0) for IES-R scores. All analyses were conducted using PASW version 19.0. Statistic Software Package (SPSS, Chicago, Illinois).

## Results

Demographics and clinical characteristics of all participants are depicted in Table 1.

**Table 1:** Demographic and clinical characteristics of participants (biofeedback and control condition) at baseline and number of sessions

	Biofeedback			Control			Test statistic^a^	*P*
	N	Median	Min–max	N	Median	Min–max		
Age, years	4	46.5	25–57	4	43.0	28–53	U = 7.00 Z = −.29	.89
Female (n, %)	4	4 (100 %)		4	2 (50 %)		*χ* ^2^ = 2.67 *df* = 1	.43^b^
IES-R	4	45	28–62	4	41.5	21–73	U = 7.00 Z = −.29	.89
Sessions completed	4	7.5	6–11	4	8.0	5–18	U = 8.00 Z = .00	1.00

^a^Mann–Whitney test was used for continuous variables. Chi square tests were used for categorical variables

^b^Fisher’s exact test

Breathing biofeedback appeared to be an easy to instruct and to implement procedure that can be complemented to TF-CBT. It was easy to instruct as patients were able to grasp the procedure with the short instruction in the first session and were able to follow the procedure during the prolonged exposure while focusing on the hotspot. The breathing exercise showed not to obstruct the patient to remain fully engaged in exposure to the traumatic event; they were able to experience high anxiety that dropped during and over the sessions. Furthermore, treatment adherence, i.e., attending sessions and performing homework was good, with all patients receiving biofeedback treatment attending all sessions, whereas from the patients receiving regular treatment, one patient did not attend one session. Consequently, the IES-R score was not filled out and was coded as missing. Another patient that was randomized to the control condition, did not respond to the treatment and was lost to treatment and therefore, the trial measurements after session 5 were missing. In this case, IES-R scores in these missing sessions were coded as missing values in the model.

Completion rates showed that all patients receiving biofeedback completed therapy within a reasonable number of sessions (median 7.5; min–max 6–11), in line with the guidelines of TF-CBT, i.e., 8–12 sessions (Creamer et al. 2004). In the TAU condition, the number of sessions was somewhat higher (median 8.0; min–max 5–18). Number of sessions completed is also depicted in Table 1.

**Fig. 1:**
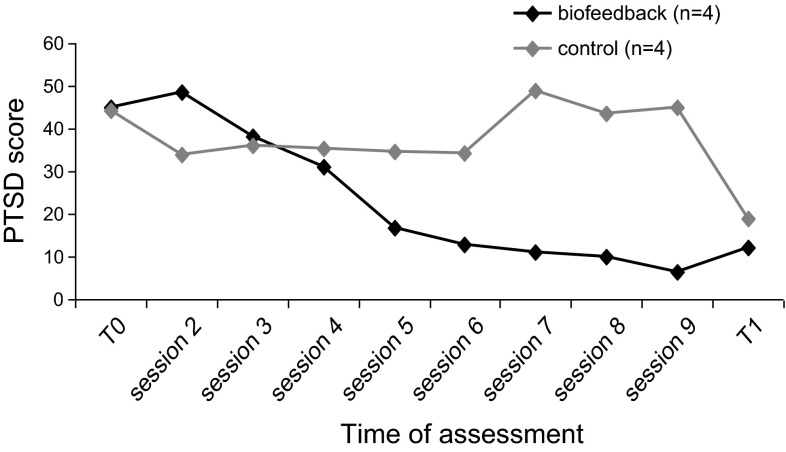
IES-R scores for biofeedback and exposure alone conditions over time

The mean PTSD symptom scores for each condition at baseline and post treatment as well as per weekly session are depicted in Fig. 1. The mixed model analysis yielded an effect for Time (*F* = 5.41; *df* = 8, *p* < .001), a trend for Condition (*F* = 4.84; *df* = 1, *p* = .061) and a borderline significant interaction for Time × Condition (*F* = 2.32; *df* = 8, *p* = .051). Although PTSD symptoms decreased over time for both conditions, PTSD symptom scores decreased faster (with borderline statistical significance) over time in the biofeedback condition.

## Discussion

This pilot study shows that breathing biofeedback is a feasible technique that can efficiently and safely be implemented during exposure therapy in PTSD patients, as it is easy to instruct to patients and easy to apprehend by patients during sessions. This is in accordance with the results of previous studies, showing that other forms of adjunct biofeedback are feasible and could efficiently and safely be implemented to CBT (Morina et al. 2012; Gevirtz and Dalenberg 2008). Most importantly, the breathing exercise showed not to obstruct the patient to remain fully engaged in exposure to the traumatic event. Notwithstanding the small sample size, a trend was clearly noticeable between conditions in favor of the biofeedback condition. More specifically, although in both conditions a significant reduction of symptoms from pre to post treatment was found, the current results show that breathing biofeedback addition exerted an additional effect through a clinically (though borderline statistically) significant faster decrease of PTSD symptoms compared to treatment as usual (TF-CBT). Interestingly, symptom decrease in the biofeedback condition occurred concurrently with the implementation of biofeedback from session 3 on (when focusing on the hotspots), and the differences between groups were more pronounced following session 3. This further suggests that it is likely that it is the biofeedback addition to the exposure element that leads to a greater decrease in PTSD symptoms. In this respect, we must note that the results may underestimate the actual additional effect of biofeedback, as we included session 1 and 2 in the analyses, despite the fact that biofeedback was not implemented yet and we only analyzed the first 9 sessions, while patients not receiving biofeedback needed more sessions. A beneficial and additional effect of biofeedback to TF-CBT compared to regular TF-CBT, is in line with previous studies of other forms of biofeedback in PTSD patients (Zucker et al. 2009; Lande et al. 2010; Tan et al. 2011). A similar faster symptom reduction was previously found in RSA biofeedback on depressive symptoms in PTSD patients as well (Zucker et al. 2009).

Several working mechanisms may be responsible for the faster PTSD symptom reduction in the biofeedback condition. First of all, the breathing component may function as relaxation, thereby directly decreasing distress and in turn inducing engagement. Indeed, previous studies support that relaxation techniques, though not advised to use as a stand-alone treatment, may to some extent decrease PTSD symptoms (Hickling et al. 1986). The beneficial effect in studies that combine biofeedback exposure (Morina et al. 2012) may be due to the combination of the effective component of exposure and the relaxation effect of biofeedback, resulting in less distress and induced engagement, and consequently a faster decrease of symptoms than exposure or biofeedback alone. Another factor that is likely to contribute even more to the reduction of PTSD symptoms may relate to the underlying working mechanism of biofeedback, namely focusing on breathing while imagining the traumatic event, may be similar to that of eye movement desensitization and reprocessing (EMDR). In EMDR, the patient has to perform eye movements while imagining the traumatic event. EMDR is shown to be an effective treatment in decreasing PTSD symptoms, and shows a faster reduction of symptoms in comparison with CBT (Nijdam et al. 2012; Ironson et al. 2002). The currently supported working mechanisms of EMDR suggest that competing working memory tasks such as eye movements may be important in reducing vividness and emotionality (van den Hout et al. 2001). Furthermore, a recent study (Engelhard et al. 2010) suggests that vividness and emotionality may be reduced due to competing working memory resources. Moreover, not only eye movements but also other taxing tasks than eye movements may reduce the vividness of the images, such as the computer game “tetris” (Engelhard et al. 2010), beeps (van den Hout et al. 2011b), calculating (Kemps and Tiggemann 2007) and drawing a complex figure (Gunter and Bodner 2008). Breathing biofeedback may be one of these taxing tasks as well, that may lead to a reduction in vividness and consecutively a reduction in PTSD symptoms. This hypothesis is supported in a recent study (van den Hout et al. 2011a), in which attentional breathing is suggested to tax working memory and reduces vividness of negative images in healthy individuals. Although we do not have any data on the vividness of the images, the attentional breathing in the current study likewise also may have reduced the vividness of negative images in PTSD patients. This mechanism of action in complementary breathing biofeedback may explain the faster symptom improvement when attentional breathing was added. This is also in line with other trials with PTSD patients showing a faster symptom reduction in EMDR in comparison with CBT (Nijdam et al. 2012; Ironson et al. 2002).

An important limitation of this study was the small sample size and therefore our results need to be interpreted with caution. In this respect, the missing values of one patient that was lost to follow-up measurement, is another drawback. Furthermore, our results are limited by the fact that breathing rates or parameters were not recorded during and over sessions in neither condition. This is particularly relevant knowing that changes in HRV and breathing do occur when symptoms in PTSD patients subside (Zucker et al. 2009), which was also the case in the control condition. Nevertheless, all patients in the TF-CBT biofeedback changed their breathing upon the feedback of the breathing device and this suggests that breathing biofeedback was the most important component for the faster symptom reduction found.

We believe our findings may be clinically relevant. First of all, only a limited number of controlled studies have been done with adjunctive therapeutic elements to regular treatment. While the feasibility of some biofeedback techniques, like RSA biofeedback and HRV biofeedback, have been studied before, this is to our knowledge the first study that focused on the feasibility of breathing biofeedback in PTSD patients. Based on the feasibility and the faster symptom improvement, even in this small sample size, it may be concluded that breathing biofeedback is a valuable complementary technique to regular TF-CBT. Future research with larger sample sizes however, could draw more definite conclusions on the effectiveness of breathing biofeedback addition to exposure in CBT and the effect on reduction in no shows and non-response in TF-CBT. Also, looking more closely into the underlying acute physiological processes and changes during breathing biofeedback may gain more insight in the exact working mechanisms involved and may provide useful directions to further improve the efficiency of this additional treatment. Future research should also examine whether adding attentional breathing to regular TF-CBT even without biofeedback would result in greater tolerance or effectiveness of exposure sessions.

In conclusion, our pilot study shows faster clinical improvement in PTSD patients receiving additional attentional breathing biofeedback. Attentional breathing biofeedback has shown a promising adjunctive element for trauma-focused CBT that can be easily implemented in clinical practice and used as a strategy for increasing efficacy of PTSD treatment.
